# A Working-Class View of Our Hospitals

**Published:** 1888-02-04

**Authors:** A. W. Webster

**Affiliations:** Chairman of Workmen's Governors, Sunderland Infirmary


### A Working-Class View of Our Hospitals.

By A. W. Webster, Chairman of Workmen's Governors, Sunderland Infirmary. (*Continued from p.* 297.)

I trust my former articles have been suggestive enough to show that it is clearly within the range of practical effort to secure substantial support for our charities from the working classes. One important section of that class, however, cannot be so easily dealt with, and some special suggestions may be given to help to bridge over the difficulties incident to their calling.

Hospital authorities in seaport towns know how large a proportion of seafaring men or their families are constantly to be found among the patients. Sailors differ from ordinary workmen inasmuch as they receive their wages at irregular periods, according to the length of their voyages, and from the very nature of their profession cannot be so effectually surrounded with influences likely to interest them in the management and systematic support of charitable institutions. An attempt has been made here to reach this class through the shipowners and shipmanagers, and although the scheme is yet in its infancy there are indications of its turning out a safe and good source of income. A large number of our local shipowners have learnt that example is better than precept, and have voluntarily imposed a tax upom themselves, so that their employers may be encouraged to contribute to hospital support. The scheme will be best understood from the following quotation, which is a copy of the matter printed on large cards, and hung up in shipping, consuls, port and dock, offices :—

"Sunderland Infirmary.

"The committee of this institution have pleasure in giving notice that seamen requiring medical and surgical aid belonging to any vessel trading to the port of Sunderland may be treated at the infirmary free of all charge, except in the case of infectious disease, for which the local authorities make special provision, and providing the case is one the surgeons consider eligible for reception as an in-patient. In order to meet, in some degree, the expense which must thus be thrown upon the institution, it has been suggested, and is very generally approved of by coal exporters, consuls, ship brokers, shipowners, and others interested, that every vessel trading to this port shall contribute to the support of the infirmary according to the following scale: Vessels not exceeding 150 tons register, 6*d*. per voyage; vessels above 150 tons and not exceeding 500 tons register, 1*s*. per voyage; vessels above 500 tons register, 2*s*. per voyage; foreign bound, over 1,000 tons register, 5*s*. per voyage. The collection of the contributions as particularised has been undertaken by the River Wear Commissioners, free of all expense, at their office, where their own port and dock charges are collected."

A hospital tax is no new imposition, for ship managers know full well that such dues are collected at almost every foreign port their ships may enter. The port and dock authorities in our gloriously free country have, I think rightly, refused to force support to their local hospitals in such a manner, yet a voluntary movement similar to the above might be initiated with little trouble and considerable profit.

As soon as the support and interest of the shipowner and manager are obtained, it is a comparatively easy matter to reach and influence the crew. Although the penny per week system can hardly be introduced here, an equivalent to it may be reached, for few sailors returning from a six weeks' voyage will refuse to contribute their sixpence, provided it be solicited at the right time and place and by the right official. I have already said that this scheme is but in its infancy here, yet I find the officers and crew of one screw collier have contributed £10 6*s*. during the past twelve months.

Paupers of one description or another are far too numerous in our country, and it is no uncommon thing to hear our most practical philanthropists charge the managers of medical and surgical institutions with helping very much in their creation. Such a charge has certainly much truth in it, in reference to the great majority of hospital out-patient departments. As a rule, very few inquiries are made of the applicants for outdoor advice and medicine, and seldom indeed is the truthfulness of their statements tested. The receipt of outdoor relief of this description has often proved the first step to a life of pauperism. Some eight or nine years ago it became very apparent to the managers of the Sunderland Infirmary that by far too large a proportion of the population was applying for outdoor medical relief. It was considered advisable to stop all dispensing and out-patient work at the infirmary, and a block of property was purchased in a central part of the town to be devoted to this purpose. A qualified dispenser was engaged, who also acted as secretary. A collector was appointed, and a code of rules drawn up to start this institution as a Provident Dispensary and Medical Association. It was intended to be as much as possible a self-supporting and separate institution from the infirmary, the only official connexion between the two being the appointing of five members of committee (out of twenty-four) to the Provident Dispensary by the infirmary authorities. An institution of this description could never be expected to become self-supporting all at once, and the Infirmary Committee granted £500 per annum (£50 of which is returned as rent) to assist in its maintenance. Admission to Sunderland Infirmary being entirely free, there are numerous applications for beds in the institution when some slight out-door treatment might be sufficient to recruit the health of the applicant. Should such persons be found temporarily destitute, dispensary tickets are granted, which make them members of the Provident Dispensary and Medical Association for a limited period. The alteration which transformed our hospital from a "ticket" to a "free" institution was almost simultaneous with the opening of the Provident Dispensary and Medical Association, the tickets formerly granted to guinea subscribers still being continued, but having the word "infirmary" changed to "dispensary."

It will be seen from this that the ticket system still remained in force as far as the out-patient department was concerned, but I am glad to say it is only nominally in existence now, as the subscribers have appreciated the change so thoroughly that they readily hand their tickets to the secretary or members of the House Committee, who have never yet had to send a suitable case away unprovided for. The founding of such an institution, semi-attached, yet independent of the infirmary, has proved eminently successful, the number of applicants for outdoor relief falling 75 per cent. the first year of its introduction, and considerably more since.

The object of the above Provident Dispensary and Medical Association is to place medical assistance within the reach of the working classes by means of their own small periodical payments. The advantages offered to those who become members may be summarised as follows :—

I. Members can be attended by one of the medical officers, whom they may choose, and if unable to go to the dispensary such attendance will be given at their own homes.

II. They will thus have a claim to medical assistance by right of their own payments, without the trouble, loss of time, and humiliation of applying to subscribers to beg a recommendation.

III. A great advantage will be derived from having prompt attention in case of illness, without the loss of time which sometimes causes very serious, if not fatal, results.

IV. Those whose occupations prevent their attendance at the dispensary may consult the medical officers at their own surgeries at the times which have been fixed by those gentlemen, with a view to meet the convenience of the working classes.

V. Membership is confined to the working classes and those of limited means, but masters or mistresses may enter their servants *ex officio* on payment of five shillings a-year, a change of servants during the year not rendering the membership invalid.

The charges for admission and monthly payments are as below: For any person under 14 years, 1*s*. 7*d*. admission and 2*d*. per month; for any person over 14 years, 1*s*. 9*d*. and 4*d*.; for an adult and one child, or three children under 14 years, 1*s*. 11*d*. and 6*d*.; for a man and his wife, 2*s*. 1*d*. and 8*d*.; for a man, his wife, and one child, 2*s*. 3*d*. and 10*d*.; for a man, his wife, and all his children under 14 years, 2*s*. 5*d*. and 1*s*.

There are seven medical officers attached to the institution, the remuneration for their services being regulated by the following rule: "Three-fifths of the payments of the provident members (with the exception of the midwifery fees) will be divided at the end of every quarter or half-year among the medical officers, in proportion to the amounts received from the members registered under their names. The balance of members' payments, together with the contri bution of the infirmary, will be appropriated towards the cost of medicines and the expenses of management. If any sum is left after defraying the expenses of management, the committee will distribute at least two-thirds of it among the medical officers."

In concluding this article I would remind my readers that the active voluntary services of workmen's representatives must be limited to their leisure hours. Any deductions made from the wages of the few who are chosen to sit on the hospital committees meeting regularly during the day should be refunded from the workpeople's subscriptions, as a class of men seldom earning more than an average of thirty shillings per week can hardly be expected to sacrifice half-a-day's pay fortnightly or monthly for any purpose whatever.

